# Prevalence and risk factors of work-related musculoskeletal disorders among emerging manufacturing workers in Beijing, China

**DOI:** 10.3389/fmed.2023.1289046

**Published:** 2023-10-12

**Authors:** Xiaowen Ding, Ziyi Guan, Nan Liu, Mingli Bi, Fang Ji, Huining Wang, Xueyan Zhang, Baolong Liu, Dongsheng Niu, Tian Lan, Tingting Xie, Jue Li, Tenglong Yan

**Affiliations:** ^1^Beijing Institute of Occupational Disease Prevention and Treatment, Beijing, China; ^2^School of Public Health, Shanxi Medical University, Taiyuan, China; ^3^Tianjin Navigation Instruments Research Institute, Tianjin, China; ^4^School of Urban Economics and Public Administration, Capital University of Economics and Business, Beijing, China

**Keywords:** work-related musculoskeletal disorders (WMSDs), worker, cross-sectional study, manufacturing workers, risk factors

## Abstract

**Objective:**

The workers in emerging manufacturing are at decreased risk of traditional occupational diseases, while probably at increased risk of work-related musculoskeletal disorders (WMSDs). This study aimed to investigate the prevalence and risk factors of WMSDs among emerging manufacturing workers in Beijing.

**Methods:**

A total of 3,359 valid questionnaires were collected from 10 enterprises in the electronics, pharmaceutical, and motor manufacturing industries. The prevalence of WMSDs was assessed using the Nordic Musculoskeletal Questionnaire. The work posture load was evaluated through a questionnaire.

**Results:**

The results showed that the highest prevalence of WMSDs was observed in part of the neck (15.0%), followed by the lower back (12.5%), shoulders (11.2%), and upper back (7.1%). Female workers, workers aged older than 35 years, workers with a BMI of ≥28 kg/m^2^, longer working experience, never exercised had a higher prevalence of WMSDs. Logistic regression analysis showed that female workers, workers aged older than 35 years, with a middle school education and college degree, and workers who never exercised had a higher risk of WMSDs. In addition, workers who sat for long during work, worked hard with upper limbs or hands, worked in uncomfortable postures, and performed repetitive operations were positively related with the increased risk of WMSDs.

**Conclusion:**

These findings suggested that WMSDs were prevalent among emerging manufacturing workers in Beijing, China, while efforts should be made to reshape the risk factors associated with WMSDs, such as prolonged sitting, uncomfortable positions, and repetitive operations. Encouraging exercise and promoting ergonomic interventions probably be also benefit to induce the risk of WMSDs.

## 1. Introduction

Work-related musculoskeletal disorders (WMSDs) refer to injuries to local muscles, tendons, bones, cartilage, ligaments, nerves, and other parts of the body caused or aggravated by occupational activities, resulting in varying degrees of damage. Approximately 1.71 billion people have musculoskeletal conditions worldwide (1). WMSDs are the leading contributor to disability worldwide, with low back WMSDs being the single leading cause of disability in 160 countries (2, 3). WMSDs significantly limit mobility and dexterity, leading to early retirement from work, lower levels of well-being and reduced ability to participate in society. Because of population growth and ageing, the number of people living with WMSDs and associated functional limitations, is rapidly increasing which has become a major occupational-related disease affecting the health of the working population. Many countries, including the United States, the United Kingdom, Germany, and Japan, had included WMSDs in their list of legally recognized occupational diseases or compensable diseases (4, 5). Therefore, it is necessary to conduct research on the prevalence and risk factors of WMSDs, which is of great significance for the prevention and treatment of WMSDs and the health protection of the working population.

Beijing, as the capital of China, is undergoing a major restructuring of its industrial structure. Currently, there are large number of workers worked in emerging industries, such as electronics, pharmaceutical, and motor manufacturing industries (6). Risk factors such as forced postures, unreasonable work systems, repetitive tasks, and long working hours were common in these industries (7, 8), which were widely presented and can easily result to local muscle fatigue and increase the risk of WMSDs (9, 10), which was significant different to the traditional risk factors, such as carry heavy objects. Electronics, pharmaceutical, and motor manufacturing industries were typical emerging manufacturing industries. The main characteristics of those enterprises were the high degree of automation and light manual labor, which was the future development direction of most enterprises. The characteristics and prevalence of WMSDs probably be different from traditional manufacturing workers, such as building industry, iron and steel industry. Therefore, it is necessary to evaluate the prevalence of WMSDs in these types of enterprises to light the other developing countries in the future. However, there are no reports on the prevalence and risk factors of WMSDs among emerging manufacturing workers in China. The objective of this study was to evaluate the prevalence of WMSDs among emerging manufacturing workers in Beijing, China. Therefore, a cross-sectional survey included 3,359 workers to determine the prevalence of WMSDs and risk factors was conducted in Beijing, which can be benefit to improve the prevention measures and occupational health status of workers, reduce the incidence of WMSDs, and alleviate the social burden.

## 2. Methods

### 2.1. Study design and participants

Ten enterprises, including electronics, pharmaceutical, and motor manufacturing industries in Beijing, China, were selected in this cross-sectional study between September 2021 and December 2022. The characteristics of each enterprises were showed in Supplementary Table S1. Among these enterprises, workers from the frontline production positions were included in this study. The inclusion criteria were as followed: (a) age more than 18 years old; (b) worked for at least 1 year in this present position; (c) volunteer to participate in this study. The exclusion criteria were that individuals who had musculoskeletal pain or discomfort in the affected area before starting the present job, those with a history of injury, and those with WMSDs caused by accident. Finally, 3,359 workers were included in this study. On the majority of working days throughout the year, these workers were on an eight-hour daily work schedule, working 5 days a week, without shifts or night shifts. Although the primary job tasks may vary, the workers were primarily engaged in frontline basic production. For example, workers in pharmaceutical companies are mainly involved in drug formulation and packaging, while those in electronic companies were responsible for product quality testing. In summary, these workers represented the most fundamental characteristics of frontline production, which were characterized by low technical complexity and high repetitiveness. This study complied with the Helsinki Declaration and was approved by the Ethics Committee of the Beijing Institute of Occupational Disease Prevention and Treatment (No. C2022006). All participants were informed and gave their consent. Due to the COVID-19 epidemic, face-to-face surveys were not feasible, so the survey was conducted online.^1^ Only one questionnaire can be submitted for per participants through technical settings. The link of questionnaire was distributed throughout the organization network to ensure that other individuals not belonged to the 10 specific enterprises do not fill out the questionnaire.

### 2.2. Independent variables

Information on the demographic characteristics and work posture load of the participants was collected through a questionnaire. (a) The demographic characteristics included gender (male and female), birth date, height (m), weight (kg), education level (junior school and below, middle school, college degree), smoking (never, seldom, sometimes, and quit smoking), drinking (never, seldom, sometimes, and quit drinking), present job tenure (years), and exercise habits (never, 1–3 times/quarter, 2–3 times/month, 1–2 times/week, and >2 times/week). The variable of age was classified into ≤35 and >35 years old. The body mass index (BMI) was calculated by the formula: BMI = weight (kg)/height squared (m^2^), which was further divided into three categories: ≤23.9, 24.0–27.9, and ≥28.0 kg/m^2^. The variable of current station was divided into four categories: <5, 5–10, 10–15, and >15 years. The work posture load mainly included information on whether the participants had long-term standing, sitting, squatting, and kneeling, carrying heavy loads, vibration, driving, repetitive work, and other adverse postures during work. The frequency of adverse posture was classified into “seldom, sometimes, often, and always.” The answer of “always” of these questions was defined as adverse postures during work, while the other answers of these questions was defined as no adverse posture during work.

### 2.3. Definition of WMSDs

The WMSDs was assessed using the Nordic Musculoskeletal Questionnaire, which mainly included information on whether musculoskeletal pain or discomfort symptoms occurred in neck, low back, shoulders, upper back, knee, wrist, leg, ankle, and elbow in the past 7 days and 12 months, the frequency of pain or discomfort, and the total duration of pain or discomfort throughout the year. The reliability and validity of this questionnaire has been tested previously, 0.87 and 0.80, respectively (11, 12). Specifically, when discomfort symptoms such as pain, stiffness, burning sensation, numbness, or tingling occur in the muscles or joints of various body parts, and meet the following criteria: (1) discomfort within the 12 months, (2) discomfort began after starting current work, (3) no accidents or sudden injuries affecting the affected area in the past, and (4) discomfort symptoms occur every month or last for more than 7 days, then it is considered as a WMSDs. Any of the following body parts: neck, lower back, shoulders, upper back, knee, wrist, leg, ankle, and elbow, with the discomfort symptoms, were defined as WMSDs. This study followed the diagnostic criteria for WMSDs established by the US National Institute for Occupational Safety and Health (NIOSH).

### 2.4. Statistical analysis

After downloading the data collected online, clean and logical error correction were performed. A database was established using Excel 2019 software for Mac. SPSS 26.0 software for Windows 10 was used for statistical analysis. Categorical variables were expressed as number (percent) [*n* (%)]. In the univariate analysis, the chi-square (*χ*^2^) test was used to analyze the differences in the prevalence of WMSDs among different industries and characteristic individuals. Furthermore, logistic regression analysis was performed to identify the influencing factors of WMSDs among the factors with statistical significance in the univariate analysis with sex, age category, BMI category, current station experience, education level, exercise frequency, work load, and categories of industries adjusted, which were found to be associated with WMSDs in single factor analysis or reported to be related with those (13, 14). A *p*-value less than 0.05 was defined statistically significant.

## 3. Results

### 3.1. Demographic characteristics

The demographic characteristics of the workers across industry categories were showed in Table 1. There were 367 (10.9%), 1,448 (43.1%), and 1,544 (46.0%) workers from electronics, motor, and pharmaceutical manufacturing enterprises, respectively in this study. Significant differences were observed in sex, age, BMI, current station, working years, education, exercise, smoking, and drinking across the three industries (*p* < 0.05). The electronics manufacturing industry had the highest proportion of male workers (86.1%), while the pharmaceutical industry had the highest proportion of female workers (54.0%). The pharmaceutical manufacturing industry had the highest proportion of workers aged 35 years and above (44.8%), while the electronics manufacturing industry had the highest proportion of workers with a college degree (83.8%). The motor industry had the highest proportion of workers who never exercised (47.1%), while the pharmaceutical manufacturing industry had the highest proportion of workers who exercised >2 times per week (12.0%).

**Table 1:** The demographic characteristics across industries categories.

Characteristics	E industry	M industry	P industry	*p*-value
*n*	367 (10.9)	1,448 (43.1)	1,544 (46.0)	—
Sex				<0.01^*^
Male	320 (87.2)	1,309 (90.4)	704 (45.6)	
Female	47 (12.8)	139 (9.6)	840 (54.4)	
Age (years old)				<0.01^*^
≤35	287 (78.2)	1,093 (75.5)	852 (55.2)	
>35	80 (21.8)	355 (24.5)	692 (44.8)	
BMI (kg/m^2^)				<0.01^*^
≤23.9	151 (41.2)	652 (45.0)	797 (51.6)	
24.0–27.9	152 (41.4)	457 (31.6)	507 (32.8)	
≥28.0	64 (17.4)	339 (23.4)	240 (15.6)	
Current station (years)				<0.01^*^
<5	198 (54.0)	614 (42.4)	809 (52.4)	
5–10	90 (24.5)	662 (45.7)	401 (22.4)	
10–15	42 (11.4)	144 (9.9)	213 (13.8)	
>15	37 (10.1)	28 (1.9)	121 (7.8)	
Education				<0.01^*^
Junior school and below	16 (4.2)	49 (3.4)	104 (6.7)	
Middle school	31 (8.1)	849 (58.6)	371 (24.0)	
College degree	320 (83.8)	550 (38.0)	1,069 (69.2)	
Exercise				<0.01^*^
Never	99 (25.9)	682 (47.1)	418 (27.1)	
1–3 times/quarter	63 (16.5)	242 (16.7)	253 (16.4)	
2–3 times/month	105 (27.5)	189 (13.1)	313 (20.3)	
1–2 times/week	61 (16.0)	253 (17.5)	374 (24.2)	
>2 times/week	39 (10.2)	82 (5.7)	186 (12.0)	

^*^p < 0.05. M industry, motor industry; E industry, electronics manufacturing; P industry, pharmaceutical industry.

### 3.2. Prevalence of WMSDs

**Figure 1:**
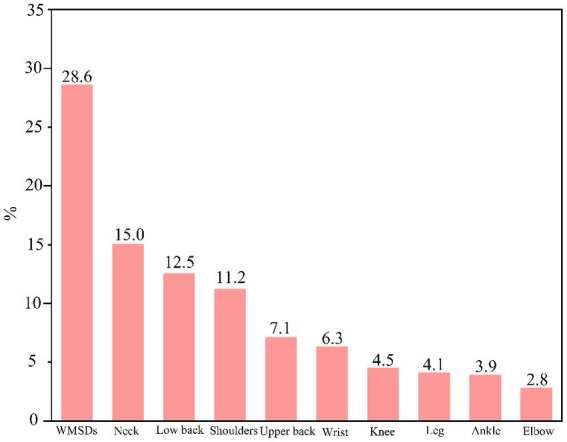
The prevalence of work-related musculoskeletal disorders (WMSDs) among workers.

The prevalence of WMSDs by body part among 3,359 manufacturing workers was showed in Figure 1. The highest prevalence of WMSDs was observed in the neck (15.0%), followed by the lower back (12.5%), shoulders (11.2%), and upper back (7.1%). The prevalence of WMSDs in the wrist and knee was 6.3% and 4.5%, respectively. The prevalence of WMSDs in the legs, ankles, and elbow was 4.1%, 3.9%, and 2.8%, respectively. In addition, approximately one-fourth of the workers suffered from at least one WMSDs in any body part.

The prevalence of WMSDs across demographic characteristics among the manufacturing workers was showed in Table 2. Significant differences were observed in the prevalence of WMSDs across sex, age, current work experience, education level, exercise, and industry categories (*p* < 0.05). The prevalence of WMSDs was higher among female workers (33.5%) than male workers (24.0%). Workers aged 35 years and above had higher prevalence of WMSDs (33.7%) than aged ≤35 years old (23.4%). Workers with the BMI of ≥28 kg/m^2^ had the highest prevalence of WMSDs (28.8%). Workers with less than 5 years of work experience had the lowest prevalence of WMSDs (22.8%), while those with 15 years or more work experience had the highest prevalence (31.8%). Workers who never exercised had the highest prevalence of WMSDs (31.2%). The pharmaceutical manufacturing industry had the highest prevalence of WMSDs (29.1%), while the electronics manufacturing industry had the lowest prevalence (24.8%).

**Table 2:** The prevalence of WMSDs across demographic characteristics.

Characteristics	Number of workers *n* (%)	WMSDs *n* (%)	*χ*^2^	*p*-value
Sex			33.187	<0.01^*^
Male	2,333 (69.5)	559 (24.0)		
Female	1,026 (30.5)	344 (33.5)		
Age (years old)			40.309	<0.01^*^
≤35	2,232 (66.4)	523 (23.4)		
>35	1,127 (33.6)	380 (33.7)		
BMI (kg/m^2^)			2.383	0.304
≤23.9	1,600 (47.6)	434 (27.1)		
24.0–27.9	1,116 (33.2)	284 (25.4)		
≥28.0	643 (19.2)	185 (28.8)		
Current work experience (years)			26.811	<0.01^*^
<5	1,621 (48.3)	370 (22.8)		
5–10	1,153 (34.3)	347 (30.1)		
>10	585 (17.4)	117 (31.8)		
Education level			22.670	<0.01^*^
Junior school or below	169 (5.0)	28 (16.6)		
Senior high school	1,251 (37.2)	299 (23.9)		
College and above	1,939 (57.8)	576 (29.7)		
Sports				<0.01^*^
Never	1,199 (35.7)	374 (31.2)		
1–3 times/quarter	558 (16.6)	147 (26.3)		
2–3 times/month	607 (18.1)	148 (24.4)		
1–2 times/week	688 (20.5)	161 (23.4)		
>2 times/week	307 (9.1)	73 (23.8)		
Categories of industries			7.445	0.02^*^
E industry	367 (10.9)	91 (24.8)		
M industry	1,448 (43.1)	362 (25.0)		
P industry	1,544 (46.0)	450 (29.1)		

^*^p < 0.05. E industry, electronics manufacturing; M industry, motor industry; P industry, pharmaceutical industry.

### 3.3. Labor load

Table 3 presented the working posture across three industries among the manufacturing workers. Significant differences were observed in the working posture across the three industries for all the nine risk factors (*p* < 0.05). The motor industry had the highest proportion of workers who stood for long periods (34.7%), while the pharmaceutical industry had the lowest proportion (8.1%). The transportation electronics industry had the highest proportion of workers who sat for long periods (30.7%), while the motor manufacturing industry had the highest proportion of workers who squatted or kneeled for long periods (3.2%). The motor and pharmaceutical industry had the highest proportion of workers who carried objects weighing more than 5 kg (7.8%) and more than 20 kg (3.3%), respectively. The motor manufacturing industry had the highest proportion of workers who worked hard with their upper limbs or hands (27.1%). The motor industry had the highest proportion of workers who were exposed to vibration (12.0%). The motor manufacturing industry had the highest proportion of workers who drove a vehicle (9.3%). The motor industry had the highest proportion of workers who worked in uncomfortable positions (6.1%). The motor manufacturing industry had the highest proportion of workers who performed repetitive operations (31.8%).

**Table 3:** The working posture across three industries.

Risk factors	E industry	M industry	P industry	*p*-value
Stand for long	41 (11.2)	503 (34.7)	125 (8.1)	<0.01^*^
Sit for long	76 (20.7)	111 (7.7)	231 (15.0)	<0.01^*^
Squat or kneeling for long	3 (0.8)	47 (3.2)	231 (0.6)	<0.01^*^
Carry objects >5 kg	10 (2.7)	113 (7.8)	106 (6.9)	<0.01^*^
Carry objects >20 kg	6 (1.6)	46 (3.2)	51 (3.3)	<0.01^*^
Working hard with upper limbs or hands	32 (8.7)	393 (27.1)	188 (12.2)	<0.01^*^
Vibration	2 (0.5)	174 (12.0)	36 (2.3)	<0.01^*^
Driving a vehicle	12 (3.3)	135 (9.3)	108 (7.0)	<0.01^*^
Uncomfortable positions	8 (2.2)	89 (6.1)	28 (1.8)	<0.01^*^
Repetitive operation	42 (11.4)	460 (31.8)	187 (12.1)	<0.01^*^

^*^p < 0.05. E industry, electronics manufacturing; M industry, motor industry; P industry, pharmaceutical industry.

### 3.4. Influencing factors of WMSDs

**Figure 2:**
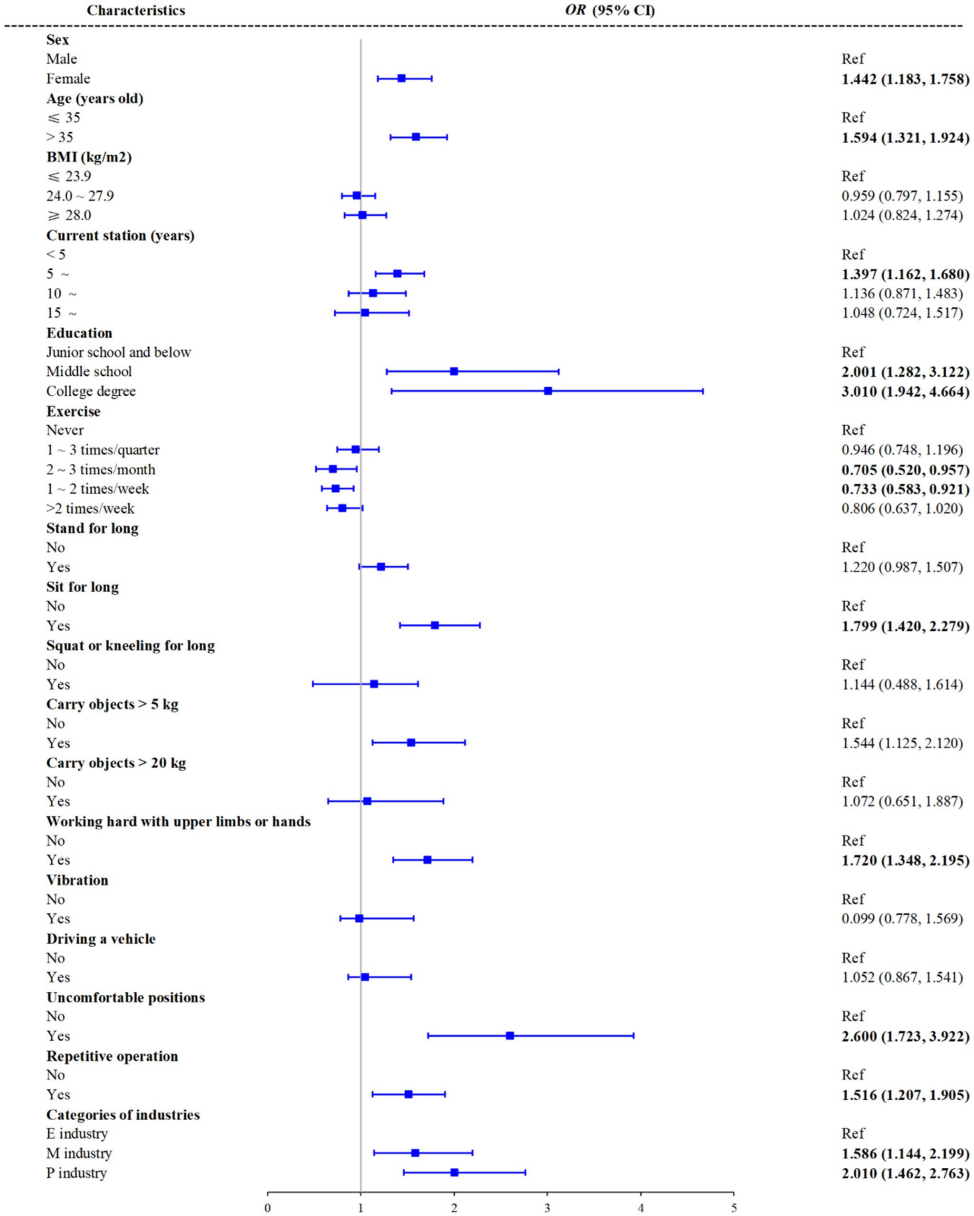
Associations between risk factors and WMSDs in multivariate logistic regression. E industry, electronics manufacturing; M industry, motor industry; P industry, pharmaceutical industry.

Univariate logistic regression was performed firstly to evaluate the association between risk factors of working posture and WMSDs, which indicated that all the postures were related with WMSDs (Supplementary Table S2). Multiple logistic regression was performed to further evaluate the association risk factors between WMSDs, which were showed in Figure 2. The associations between WMSDs and sex, age, current station, education level, exercise, sit for long, working hard with upper limbs or hands, uncomfortable positions, and repetitive operations were statistically significant (*p* < 0.05). Female workers had a higher odds ratio (OR) of WMSDs (OR = 1.442, 95% CI: 1.183, 1.758) than that among male workers. Workers aged 35 years old and above had higher ORs of WMSDs than those aged under 35 years old (*p* < 0.05). Workers with a middle school education and college degree had higher ORs of WMSDs than those with a junior school education or below (*p* < 0.05). In addition, exercise was found to be a protective factor to WMSDs. Workers who exercised 2–3 times per month and 1–2 times per week had lower ORs of WMSDs than those who never exercised (*p* < 0.05). Workers who sat for long during work had a higher OR of WMSDs (OR = 1.632, 95% CI: 1.332, 1.999) than those who did not. Workers who worked sitting for long, with upper limbs or hands, in uncomfortable positions, and performed repetitive operations had higher ORs of WMSDs than those who did not (*p* < 0.05). No significant association was observed between WMSDs and standing for long periods, kneeling or squatting for long periods, carrying objects weighing more than 20 kg, vibration, or driving a vehicle (*p* > 0.05).

## 4. Discussion

This study aimed to investigate the prevalence and risk factors of WMSDs among emerging manufacturing workers in Beijing, China. The results showed that the prevalence of WMSDs was high among the workers, with the neck, lower back, shoulders, and upper back being the most commonly affected body parts. Female workers, older workers, workers with higher education levels, and those who never exercised were more likely to suffer from WMSDs. In addition, sitting for long periods, working in uncomfortable positions, and performing repetitive operations were identified as risk factors for WMSDs.

The prevalence of WMSDs in this study was 28.6%, which was lower than that among 7,908 manufacturing workers in Henan and Hubei Provinces, China (10), consistent with that among workers in manufacturing factories in Guangdong Province, China (15). The WMSDs prevalence of the neck, low back, shoulders, and upper back was the highest among all the body parts, which was consistent with other studies (16, 17). The work of manufacturing often involved risk factors of repetitive tasks, prolonged sitting or standing, and awkward postures, which could lead to local muscle fatigue and increase the risk of WMSDs (5). Female workers were found to be more likely to suffer from WMSDs than male workers, which is consistent with previous studies (5, 18). Women generally have smaller muscle mass and lower strength than men, which may make them more vulnerable to WMSDs (5). In addition, women were more likely to work in jobs that require repetitive tasks and prolonged standing or sitting, which are risk factors for WMSDs (19, 20). The older workers were also found to be more susceptible to WMSDs than young workers, which was consistent with previous studies (21). This may be due to age-related changes in the musculoskeletal system, such as decreased muscle strength and flexibility, soft tissue rheumatism, osteoarthritis, inflammatory arthritis, large joint prostheses, and age-related co-morbidities, which can increase the risk of WMSDs (21, 22). In addition, older workers may have accumulated more work-related physical stress over time, which can also increase the risk of WMSDs (18, 23). Furthermore, workers with higher education levels were found to be more likely to suffer from WMSDs (24), which probably be resulted to the fact that workers with higher education levels were more likely to work in jobs that require prolonged sitting, breaks less, which were risk factors for WMSDs (17). In addition, high workloads probably be another reason to increase the risk of WMSDs (25). Sitting for long periods, working in uncomfortable positions, and performing repetitive operations were identified as risk factors for WMSDs, which was consistent with previous studies (5, 8). These factors can lead to local muscle fatigue and increase the risk of WMSDs (5). Therefore, it is important to implement ergonomic interventions, such as adjusting workstations, providing rest breaks, and rotating tasks, to reduce the risk of WMSDs (8). As for the factors of work posture load, sitting for long periods, working in uncomfortable posture, and performing repetitive operations were identified as risk factors for WMSDs, factors of which could lead to local muscle fatigue and increase the risk of WMSDs (26–29). Therefore, it was crucial to implement ergonomic interventions, such as adjusting workstations, providing rest breaks, and rotating tasks, to reduce the risk of WMSDs.

Exercise was found to be a protective factor for WMSDs, which was consistent with previous studies (30, 31). Exercise can improve muscle strength and flexibility, reduce fatigue, and prevent WMSDs (32, 33). Workers who never exercised had the highest prevalence of WMSDs, while those who exercised 2–3 times per month and 1–2 times per week had lower odds of WMSDs, which suggested that even moderate levels of exercise can be beneficial for preventing WMSDs.

However, it was not easily to engage in physical exercise, even if it was only mild exercise, which was benefit to prevent the occurrence of WMSDs.

To the best of our knowledge, this is the first study to illustrated the prevalence of WMSDs among the emerging manufacturing enterprises workers and to explore the risk factors in large samples in Beijing, China. The current study illustrated that workers in the emerging manufacturing enterprises were at high risk of WMSDs although whose labor load was not as strong. Work load risk factors of siting for long, carry objects, uncomfortable positions were associated with the risk of WMSDs, while the demographic and habits of female, age, educational level, and sports were also associated with the happen of WMSDs. Therefore, ergonomic interventions, including implement ergonomic interventions to address work load risk factors such as prolonged sitting, carrying heavy objects, and uncomfortable positions, could be applied in the workplace. In addition, providing training and education programs to raise awareness among workers about the importance of maintaining good posture, using proper body mechanics, and adopting ergonomic practices probably be benefit to reshape the WMSDs, which can help them understand the risks associated with WMSDs and learn preventive measures. By implementing these measures, it is possible to reduce the prevalence of WMSDs among workers in emerging manufacturing enterprises and improve their overall musculoskeletal health.

This study had notable strengths due to its large sample size. Nevertheless, it was important to acknowledge several limitations that were present in this study. First, the study was conducted based on a cross-sectional design, which limited the ability to establish causality between risk factors and WMSDs. Second, the study relied on self-reported data, which may be subject to recall bias and social desirability bias. There may be inaccuracies when participants recalled WMSD or not over a year. In addition, some participants tended to exaggerate the severity of WMSDs when they were dissatisfied with the enterprises. Third, only three types of industries workers in Beijing were included, especially the disproportionally small sample size of the electronics group, which may limit the generalizability of the findings to other regions and industries in China and other regions. Forth, the data were collected online since the epidemic of COVID-19, while the information bias was common for that. Therefore, it is necessary to further verify the conclusions of this study.

## 5. Conclusion

This study provides important insights into the prevalence and risk factors of WMSDs among manufacturing workers in Beijing, China. The high prevalence of WMSDs and the identified risk factors highlight the need for targeted prevention measures, such as ergonomic interventions and exercise. In addition, there is currently a lack of objective and reliable diagnostic methods for WMSDs. It is necessary to conduct research in the future to address this gap.

## Data availability statement

The raw data supporting the conclusions of this article will be made available by the authors, without undue reservation.

## Ethics statement

The studies involving humans were approved by the Medical Ethics Committee of the Beijing Institute of Occupational Disease Prevention and Treatment. The studies were conducted in accordance with the local legislation and institutional requirements. The participants provided their written informed consent to participate in this study.

## Author contributions

XD: Conceptualization, Formal analysis, Methodology, Writing – original draft. ZG: Data curation, Methodology, Software, Writing – review & editing. NL: Supervision, Writing – original draft. MB: Data curation, Investigation, Methodology, Writing – review & editing. FJ: Supervision, Writing – review & editing. HW: Supervision, Writing – review & editing. XZ: Project administration, Writing – original draft. BL: Resources, Supervision, Writing – review & editing. DN: Supervision, Writing – review & editing. TL: Project administration, Resources, Writing – original draft. TX: Project administration, Writing – original draft. JL: Funding acquisition, Supervision, Writing – review & editing. TY: Conceptualization, Funding acquisition, Investigation, Resources, Supervision, Writing – original draft, Writing – review & editing.

## Footnotes

^1^https://www.wjx.cn/newwjx/manage/myquestionnaires.aspx

## Abbreviations

WMSDs: Work-related musculoskeletal disorders
OR: Odds ratio
CI: Confidence interval
