# Supplementary material for: Prevalence and risk factors of work-related musculoskeletal disorders among emerging manufacturing workers in Beijing, China
Source: Front Med (Lausanne). 2023 Oct 12;10:1289046. doi: 10.3389/fmed.2023.1289046 (PMC10602678; doi:10.3389/fmed.2023.1289046)
Supplement: Supplementary file 1 [file Table_1.DOCX]

**Table S1.** The characteristics of participating corporations.

| **Corporation no.** | **Number of workers (*n*)** | **Industry category** |
| --- | --- | --- |
| 1 | 1448 | Motor industry |
| 2 | 90 | Electronics manufacturing |
| 3 | 277 | Electronics manufacturing |
| 4 | 451 | Pharmaceutical industry |
| 5 | 295 | Pharmaceutical industry |
| 6 | 384 | Pharmaceutical industry |
| 7 | 133 | Pharmaceutical industry |
| 8 | 52 | Pharmaceutical industry |
| 9 | 46 | Pharmaceutical industry |
| 10 | 183 | Pharmaceutical industry |
| Total | 3359 | — |

M industry: Motor industry; E industry: Electronics manufacturing; P industry: Pharmaceutical industry; T industry.
