# Supplementary material for: Prevalence and risk factors of work-related musculoskeletal disorders among emerging manufacturing workers in Beijing, China
Source: Front Med (Lausanne). 2023 Oct 12;10:1289046. doi: 10.3389/fmed.2023.1289046 (PMC10602678; doi:10.3389/fmed.2023.1289046)
Supplement: Supplementary file 2 [file Table_2.DOCX]

**Table S2**. Associations between risk factors and WMSDs in univariate logistic regression.

| Characteristics | *OR* | *OR* 95% *CI* | *p value* |
| --- | --- | --- | --- |
| Stand for long | 1.309 | 1.091, 1.570 | 0.004 ^*^ |
| Sit for long | 2.315 | 1.877, 2.854 | < 0.01 ^*^ |
| Squat or kneeling for long | 1.931 | 1.152, 3.235 | 0.013 ^*^ |
| Carry objects > 5 kg | 2.471 | 1.885, 3.241 | < 0.01 ^*^ |
| Carry objects > 20 kg | 2.629 | 1.773, 3.897 | < 0.01 ^*^ |
| Working hard with upper limbs or hands | 2.317 | 1.933, 2.777 | < 0.01 ^*^ |
| Vibration | 1.925 | 1.450, 2.555 | < 0.01 ^*^ |
| Driving a vehicle | 1.618 | 1.243, 2.108 | < 0.01 ^*^ |
| Uncomfortable positions | 3.969 | 2.752, 5.724 | < 0.01 ^*^ |
| Repetitive operation | 2.196 | 1.844, 2.614 | < 0.01 ^*^ |
